# Selective Mid-IR Metamaterial-Based Gas Sensor System: Proof of Concept and Performances Tests

**DOI:** 10.3390/nano12061009

**Published:** 2022-03-18

**Authors:** Laura Mihai, Razvan Mihalcea, Roxana Tomescu, Costel Paun, Dana Cristea

**Affiliations:** 1 National Institute of Laser, Plasma and Radiation Physics—INFLPR/CETAL, 077125 Ilfov, Romania; laura.mihai@inflpr.ro (L.M.); razvan.mihalcea@inflpr.ro (R.M.); 2 National Institute for Research and Development in Microtechnologie—IMT Bucharest, 077190 Ilfov, Romania; costel.paun@imt.ro (C.P.); dana.cristea@imt.ro (D.C.)

**Keywords:** metamaterials, metasurfaces, perfect absorber, IR selective source, gas sensing system

## Abstract

In this paper, we propose a highly selective and efficient gas detection system based on a narrow-band IR metasurface emitter integrated with a resistive heater. In order to develop the sensor for the detection of specific gases, both the microheater and metasurface structures have been optimized in terms of geometry and materials. Devices with different metamaterial structures and geometries for the heater have been tested. Our prototype showed that the modification of the spectral response of metasurface-based structures is easily achieved by adapting the geometrical parameters of the plasmonic micro-/nanostructures in the metasurface. The advantage of this system is the on-chip integration of a thermal source with broad IR radiation with the metasurface structure, obtaining a compact selective radiation source. From the experimental data, narrow emission peaks (FWHM as low as 0.15 μm), corresponding to the CO_2_, CH_4_, and CO absorption bands, with a radiant power of a few mW were obtained. It has been shown that, by changing the bias voltage, a shift of a few tens of nm around the central emission wavelength can be obtained, allowing fine optimization for gas detection applications.

## 1. Introduction

**Figure 1:**
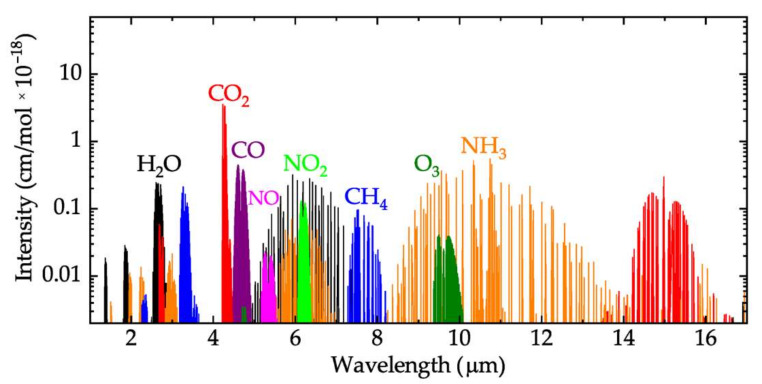
The spectral features for the main indoor and outdoor harmful gases [14].

The development of highly selective, sensitive, compact, and low-cost sensors for the detection and monitoring of harmful gases is of high interest for the health, safety, security, and environment sectors [1,2,3,4,5,6,7,8]. Rapid modernization, industrialization, but also the prolonged time spent inside in the last period (e.g., homes, offices, laboratories) are important sources of hazardous gas emission in the air [9,10,11,12,13]. Worldwide, there are about 7 million premature deaths a year from air pollutants (indoor and outdoor pollutants) [10,11]. Some of the most significant pollutants are carbon monoxide (CO), nitrogen dioxide (NO_2_), and carbon dioxide (CO_2_) from air ventilation (indoor sources) and ground-level ozone (O_3_) (outdoor source). Greenhouse gases, such as carbon dioxide (CO_2_), methane (CH_4_), nitrous oxide (N_2_O), also have an important impact on the environment and human health [11,12,13]. One of the most used techniques for the detection, identification, and monitoring of different gas molecules in a mixture is infrared spectroscopy in the mid-infrared (Mid-IR) spectral range. This is because the targeted gases mentioned above have their absorption features in this area of the electromagnetic field [14,15,16,17,18,19], as one can see in Figure 1. Mid-IR gas sensor types are different in cost and efficiency, depending on their configuration [16,17,18,19,20,21,22,23,24,25,26,27,28]. Some of the most sensitive systems (sensitivity up to parts-per-trillion concentration), such as the mid-infrared sensor system based on continuous-wave (CW) interband cascade laser (ICL) [16], or those based on Tuneable Diode Laser Absorption Spectroscopy (TDLAS), Quartz-Enhanced Photoacoustic Spectroscopy (QEPAS) [15], include laser as emitting sources. Their disadvantages are the high cost (e.g., the price for a QCL laser is high) and their dimensions (they are generally are benchtop setups). Another problem for these types of sensors and for gas sensors in general is related to their selectivity capabilities, which is important when multiple gases with overlapped absorption spectra have to be identified (e.g., around 7.5 µm; see Figure 1).

Recent studies have reported as an alternative to laser-based gas sensors IR quasi-monochromatic sources that use “perfect” absorbers based on metamaterial [20,21,22,23,24,29,30]. The advantage of this type of IR source is the wavelength selectivity, having the possibility to be tuned within UV up to the THz range, only by changing the type of material and the geometrical configuration [22,25,26,27]. Besides that, the compatibility of metamaterials with classic fabrication processes (such as photolithography and lift-off) has been proved recently, minimizing the production costs [31]. This kind of IR emitter is based on a metal–dielectric–metal (MDM) metamaterial used as a perfect absorber/narrow-band emitter and a thermal source having the role of broadband IR source [32,33]. Generally, the thermal source is external. The MDM structure consists of a metamaterial with periodical or random micro-/nanostructures, a dielectric, and a back reflector. The control of the spectral emissivity/absorptivity is achieved with geometrically tuned electromagnetic resonances of the nanostructures.

In this work, we propose and demonstrate the functionality of a highly selective and efficient gas sensor based on a narrow-band IR metamaterial emitter integrated with a resistive heater, having different configurations for multiple gas detection. In order to optimize the sensor for the detection of specific gases, the structure and the geometry of both the microheater and the metamaterial have been optimized. Details related to system design, fabrication processes, and characterization methods are presented in Section 2. Our prototype showed that the modification of the spectral response of the metasurface-based structures is easily obtained by tailoring the geometrical parameters of the plasmonic micro-/nanostructures from the metasurface.

The advantages of this system as compared with those in the literature are: (i) the on-chip integration of an IR broad thermal source with the metasurface structure obtaining a compact selective radiation source, (ii) the presence of an amorphous silicon layer on top of the metasurface structure offering an optimum heat transfer between the two components, and (iii) the proposed IR source can be easily designed to have a narrow emission adapted to the target gas absorption spectra, thus ensuring a very good selectivity.

## 2. Sensor Structure and Technology Process

**Figure 2:**
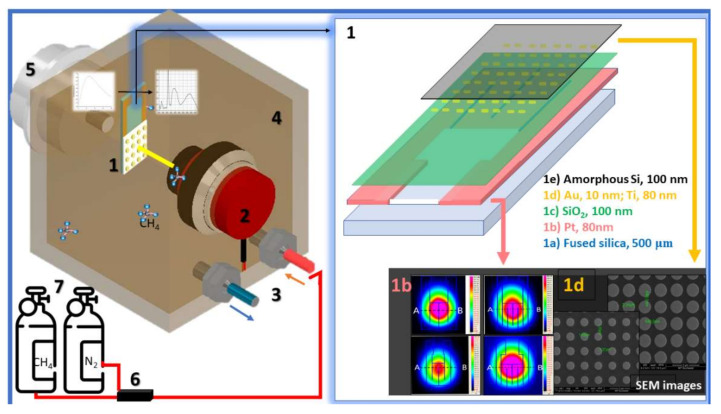
Conceptual diagram of the proposed gas sensing platform based on metamaterial structures integrated with a microheater. The sensor assembly is composed of: a (**1**) micro-IR source based on a metamaterial “perfect” absorber integrated with a microheater for selective gas sensing, which has the property to change the broadband emission of the microheater into a narrow and high-intensity emission band with the center wavelength overlapping the absorption peak of test gases; (**2**) thermopile detector; (**3**) two gas plug-ins (inlet and outlet); (**4**) gas chamber, where a mixture of an inert gas (N_2_) and a test gas is purged; (**5**) manometer; (**6**) mass flow controller for mixing nitrogen with toxic gases (e.g., CH_4_, CO_2_) in different concentrations; and (**7**) inert and test gases tanks. The narrow-band IR source (**1**) is composed of 5 layers: (**1a**) substrate, (**1b**) Pt heater (inset—thermal images obtained with an IR camera), (**1c**) SiO_2_ insulator, (**1d**) metasurface based on Au nanodisks (inset—SEM image), and (**1e**) amorphous Si.

A schematic drawing of the proposed highly selective Mid-IR metamaterial-based gas sensor system is illustrated in Figure 2. The sensor setup includes: (1) an IR emitter based on a metamaterial “perfect” absorber (MPA) integrated with a microheater, (2) a thermophile detector, and (3) a gas chamber (with the corresponding gas plug-ins (4) and manometer (5)). The sensor working principle is the following: the resistive microheater biased with a DC voltage will generate a broadband thermal radiation similar to blackbody radiation.

The integrated metamaterial is designed to exhibit perfect absorption of the IR radiation in a specific narrow band fitted to the absorption features of the target gas. At equilibrium, the emissivity of the material equals its absorptivity (Kirchhoff’s law of thermal radiation), so the metamaterial will radiate energy in the same narrow spectral range. This radiation reaches the sensitive area of a thermopile detector that is aligned with the center of the micro-IR emitter active area. Before inserting the target gas inside the cavity, the radiant power emitted by the micro-IR source is measured using the thermopile detector. This signal is considered the background signal. The target gas is purged in different mixtures into the gas chamber with the aid of a dedicated setup, which allows the barbotage of various gases and vapors; the target is absorbing the emitted radiation (that is fitted to its spectral characteristics), decreasing the optical power that reaches the detector. The principle of operation is based on the concentration-dependent absorption of the photons at a specific gas wavelength. The measured signal when the target gas is purged into the cavity is named sample signal. The target gas concentration is established with a dedicated software that controls the target and inert (N_2_) gas debit. The sensor proof of concept for different gases is presented in Section 3.3.

The structure of the micro-IR source is shown in Figure 2, Section 1. The technology process consists of the following steps:

The first step is the fabrication of the microheater. The process is the following: an 80 nm thick platinum layer (II) is deposited by electron beam evaporation method on a 500 µm thick fused silicon wafer (I) and patterned to obtain the resistive heater that generates a broadband thermal radiation. For this work, four different models for the resistive heater have been proposed: (i) two-meander model and (ii) three-meander model, both with (a) large and (b) narrow contact pads (Figure 2, Section 1b). From Figure 2, Section 1b, one can see that the best option in terms of maximum temperature on the emitter surface is the two-meander model with narrow pads. The microheater’s active area is 1 cm^2^, the distance between two consecutive meanders is 10 μm, and the width of a meander is 2490 μm for the two-meander pattern and 1660 μm for the three-meander pattern. The distance to the contact pads is 1 cm. The platinum layer also acts as a back reflector for the selective IR source. The patterning methods used to obtain the desired configuration for the resistive layer were photolithography and lift-off. A detailed description of the microheater configuration, fabrication, and thermal characterization is presented in the paper of Paun et al. [33]. The temperatures in the center of the active area of the heater were measured using a thermocouple type-K connected to an AX-5002 Axiometer. This equipment is capable of recording temperature values in the range of 100 ÷ 1300 °C with an accuracy of 0.1%. Current–voltage curves, radiant power as a function of different applied DC bias voltages, spectral radiance, and surface temperature distribution were measured for all four microheater configurations. Radiant power was measured using a high-sensitivity thermal sensor. The thermal sensor is sensitive in the spectral range of 0.19–20 µm and power range of 8 µW–3 W [3]. The spectral radiance was measured with a complex radiometric system based on a dual monochromator and a high-sensitivity nitrogen-cooled HgCdTe detector. This system can measure the spectral radiance of a source in the spectral range of 0.25–20 µm with a spectral resolution down to 0.1 nm (the best value). The spectral emittance of the heather was determined as the ratio between the spectral radiance of the heater to the spectral radiance of the laboratory standard blackbody (BB) source type. The beam from both sources (microheater and BB) was focused on using a collimating mirror system at the double monochromator input. The BB is adjustable in the temperature range of 100–1200 °C and has an emissivity of 0.99 ± 0.01 and an uncertainty of ±2 °C in the digital temperature readout. The characterization results are presented below in Section 3.1.

To obtain the integrated narrow-band source, the process is continued with the following steps:

A thin dielectric layer (III; Figure 2(1c)) was deposited on the microheater to ensure the electrical isolation between the plasmonic metasurface (Au pillars) and the Pt heater. The heater pads have to be opened to allow electronic contact with the external wires. The first selection for the dielectric material was Al_2_O_3_ deposited with atomic layer deposition (ALD) method at a low temperature in order to obtain a soft thin film, which permits the lithographic process for opening the microheater pad area for electrical contact. The tests showed that around the temperature needed to heat the resistive material (300 °C), the deposited Al_2_O_3_ layer exfoliates, losing contact with the heater. Al_2_O_3_ is a ceramic material and is really hard to pattern once deposited at a higher temperature. The best method to pattern this insulator is the employment of a shadow mask during the deposition process. Since no optimal material could be found for this contact mask that would withstand the high deposition temperature, not allowing Al_2_O_3_ to enter underneath the mask, the ALD-deposited Al_2_O_3_ layer was replaced with a SiO_2_ film. The deposition of a SiO_2_ layer was performed at 700 °C using the low-pressure chemical vapor deposition (LPCVD) method. This dielectric layer was patterned (to open the pads) using photolithography and wet etching. The measurements showed that the SiO_2_ layer deposited at a temperature higher than the working temperature of the heating resistor does not exfoliate during the operation of the Pt resistor. Therefore, in the final configuration, a 100 nm thick SiO_2_ layer was deposited on top of the Pt resistor and patterned for opening the pads.

The metamaterial perfect absorber (MPA (IV)) layer consisting of a rectangular array of subwavelength gold cylindrical unit cells (Figure 2, Section 1d) was deposited on the dielectric layer and patterned using photolithography and lift-off method. Several geometrical configurations for the microresonators (cylinder diameters (d) and periods (P)) were numerically investigated using finite-difference time-domain (FDTD) method provided by OptiFDTD commercial software from OptiWave Canada to obtain absorption spectra that overlap with the absorption characteristics of the target gases. The MPA simulation conditions we used to determine a configuration for a metasurface that offers improved absorption on narrow wavelength intervals are detailed in [34]. The same simulation parameters were employed here; the only difference is the diameter of the metallic disks that form the metamaterial, which are at a microscale. The optimal combinations obtained for the detection of toxic gases (CH_4_, NO_2,_ NO, etc.) are shown in Table 1.

**Figure 3:**
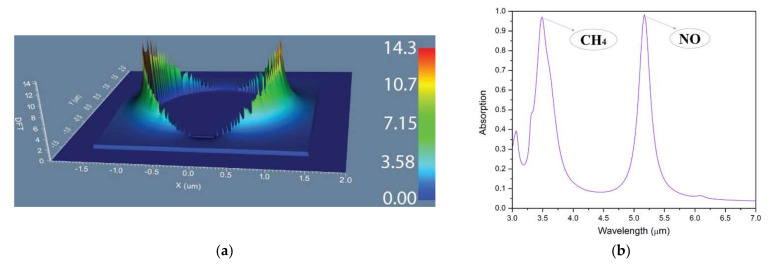
Simulation results for a metasurface composed of gold cylindrical resonators with a 2 µm diameter placed with a period of 3 µm: (**a**) electromagnetic field diagram around a metallic resonator; (**b**) absorption spectra.

Figure 3 shows an example of the electromagnetic field distribution around the metallic resonator (Figure 3a) with a 2 µm diameter placed with a period of 3 µm in a metasurface configuration and the absorption spectra (Figure 3b). To investigate the presence of resonant modes, we performed 3D-FDTD simulation using a CW at 5.1 µm wavelength (one of the absorption peaks obtained with this configuration). From Figure 3a, one can see that the metallic structures have a dipole behavior presenting clearly high resonant mods. From Figure 3b, we can conclude that this configuration presents absorption peaks at specific wavelengths and narrow intervals, making this configuration optimal for CH_4_ or NO detection. 

The spectral absorptivity was determined from energy conservation law, A = 1-T-R, taking into account that the material transmittance T is approximatively zero due to the back-reflection Pt layer. Thus, the absorptivity depends only on material reflectance R, A = 1-R.

**Figure 4:**
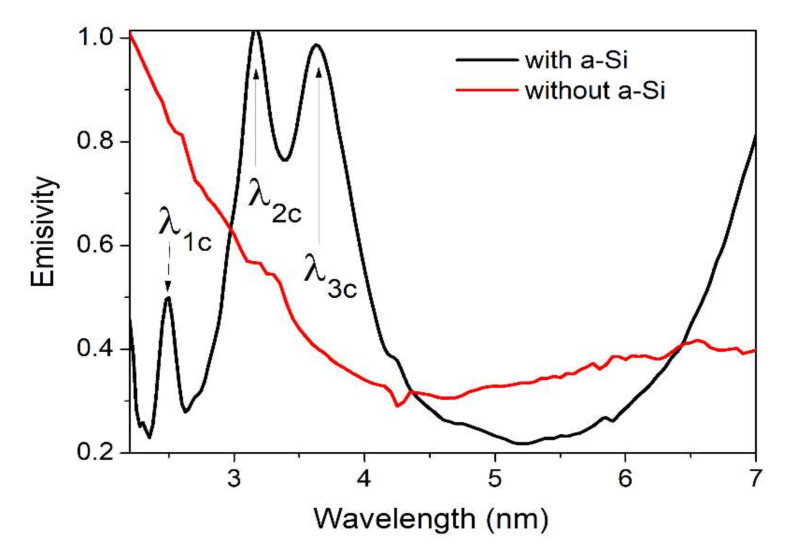
Comparison between the emissivity spectra of a selective source consisting of a platinum microheater in a three-meander configuration with wide pads and a metamaterial formed by an array of 2 µm diameter circular resonators placed at a 4 µm period, with and without amorphous Si. The peak central wavelengths: λ_1c_ = 2.5 µm; λ_2c_ = 3.174 µm; λ_3c_ = 3.633 µm, and their corresponding full width half maximum: FWHM1 = 144.06 nm; FWHM2 = 156.27 nm; FWHM3 = 279.68 nm.

The following step in the fabrication process was the deposition of the amorphous Si layer (Figure 2(1e)) to improve the spectral characteristics of the emitter. To avoid the possible degradation of this layer during the photolithography applied for the metamaterial, it was checked by simulations whether this layer could be deposited at the end of the process on top of the metamaterial structure without affecting the microheater characteristics. Both simulations and measurements showed that the deposition of the amorphous Si layer on top of the metallic metamaterial microstructures (Au/Ti with Ti as an adhesion layer) improves the spectral characteristics of the microheater, allowing for achieving higher selectivity, better defined as maximum and narrower emission band. Finally, the pads were opened in the amorphous silicon by the photolithography and etching processes using a photoresist layer as a lithographic mask. The radiant power of the selective emitter, with and without the amorphous Si layer, was measured, and the results showed that after deposition of the amorphous Si layer, the emitted power increases. This can also be seen in Figure 4, which shows the spectral emissivity of the same structures with and without Si amorphous deposition on top of the metamaterial.

## 3. Characterization Methods; Results and Discussion

The IR emitter (Figure 2(1)) was spectrally characterized using the radiometric method mentioned above, comparing the spectral radiance of the proposed IR emitter with the spectral radiance of the blackbody (BB, the laboratory standard for emissivity), set to have the same temperature. The radiant power as a function of applied DC voltage was measured using the high-sensitivity thermal sensor that was used for the microheater. The beam profile was measured with a beam-profiler-based camera sensitive in the spectral range of 2–16 µm. The detection area of the beam profiler is a silicon microbolometer. All the results obtained for the characterization of all the proposed configurations for the narrow-band IR emitter are presented and discussed in Section 3.

### 3.1. Microheater Capabilities

**Figure 5:**
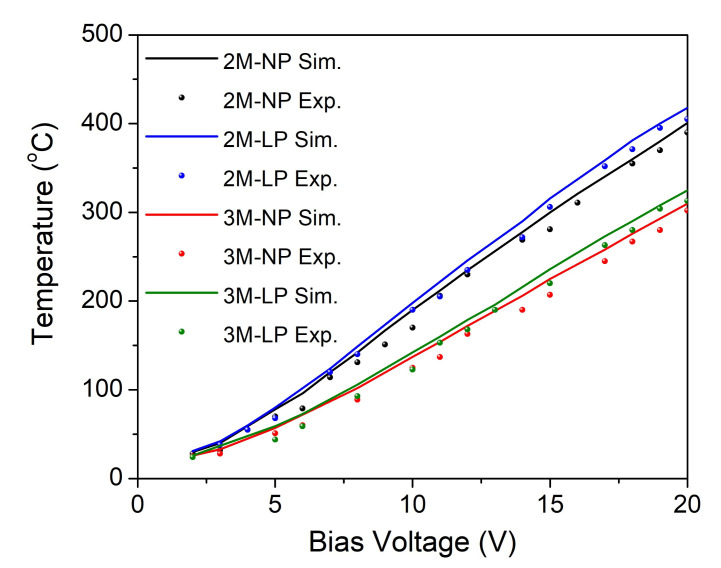
Temperature changes as a function of applied bias voltage (from 0 to 20 V) for microheaters having: two meanders and narrow pads (2M-NP) (black), three meanders and narrow pads (3M-NP) (red), two meanders and large pads (2M-LP) (blue), and three meanders and large pads (3M-LP) (green). Comparison between simulation (Sim.) and experimental (Exp.) data.

The results of the thermal characterization for all four proposed microheaters are shown in Figure 5. Here, the temperature variation as a function of the applied bias voltage for each microheater was registered in the center of the active area and compared with the simulations results. As can be seen in Figure 5, a very good correlation between simulation and experiments was obtained.

**Figure 6:**
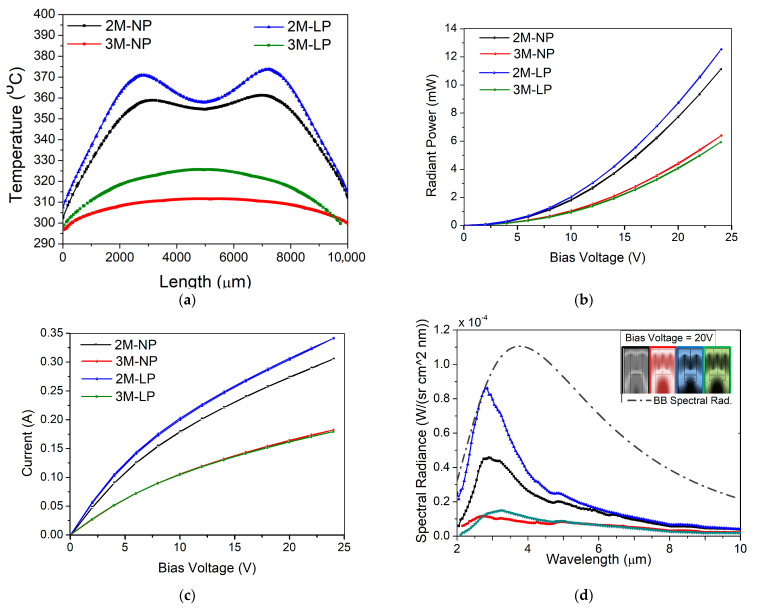
Characterization of four different microheater structures with: two meanders and narrow pads (2M–NP) (black), three meanders and narrow pads (3M–NP) (red), two meanders and large pads (2M–LP) (blue), three meanders and large pads (3M–LP) (green). (**a**) Temperature distribution, (**b**) radiant power, (**c**) I–V curve when a bias voltage of 0–24 V is applied, and (**d**) spectral radiance at 20 V.

The minimum temperature value that is requested by the metamaterial structure to be emissive is 300 °C; it can be reached by all proposed microheater configurations when a minimum bias voltage of 20 V is applied. The variation of temperature values over the sensor surface was also measured, this being a good indicator for the heater performance and endurance in time [22]; the results are presented in Figure 2(1b) and Figure 6a. We can see that for the configurations having two meanders, if a DC voltage of 20 V is applied on the pads, we obtain a very good heat distribution (360–370 °C over the central area of 6 mm and above 300 °C for the entire surface), showing the feasibility of these heaters for IR emitter devices. These results correlated with the optical characteristics are represented in Figure 6. The microheater geometry with two meanders proved to have the best performance in terms of radiant power, up to 12.55 mW (see Figure 6b), in comparison with the three-meander configuration that reaches a maximum radiant power of 6.41 mW (Figure 6b).

Figure 6c presents the I–V curve when a bias voltage between 0 and 24 V is applied. Figure 6d shows the spectral radiance curve for each heater geometry against the spectral blackbody radiance, measured for a temperature of 496.5 °C (769.65 K). This temperature value was reached by the two-meander and large-pad configuration when 20 V was applied. In any case, the spectral radiance for all the configurations proposed here is uniform, with a broadband spectrum, having maximum values between 2 and 4 μm, covering thus the spectral range in which the gas molecules of our interest have their spectral footprint.

### 3.2. Metamaterial IR Selective Emitter Capabilities

**Figure 7:**
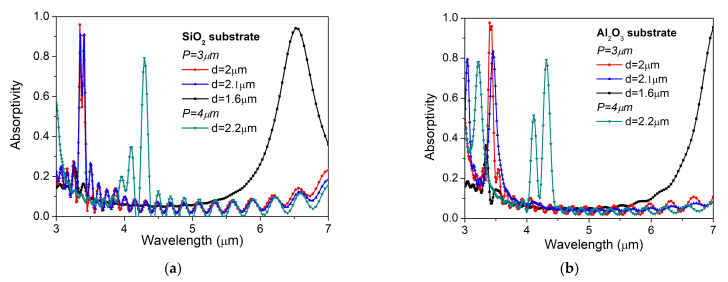
Differences between absorption spectra corresponding to IR emitter with (**a**) SiO_2_ versus (**b**) Al_2_O_3_ dielectric layer for several metasurfaces with different structures (P, metasurface period; d, disk diameter).

The selective IR emitter was obtained by narrowing the emission spectral range of the microheater presented in Section 3.1 by adding on the top of it a gold MPA structure. From our knowledge, this is one of the first systems with the metamaterial configuration integrated with the resistive heater. Two types of tunability have been proved for the proposed IR source: a coarse selectivity (hundreds of nm), changing the metamaterial geometry (gold resonator diameter and period), and a fine tuning (of few nm) by changing the bias voltage. The design and fabrication of an MPA are described in a paper [33]. The optical and electrical performances for each combination (heater and MPA) were tested and are presented in this section. As presented in Section 2, the dielectric layer used for the MDM structure was SiO_2_ deposited at 700 °C. We performed several numerical analyses to check whether this layer can offer good results in terms of absorption spectra. The simulation results showed that the absorption features for the structure with SiO_2_ are similar with the structures using Al_2_O_3_ as dielectric. Moreover, in Figure 7 one can see that SiO_2_ structures offer higher intensity, close to 1, and narrower absorption peaks in the spectral range where gases of interest (CH_4_, CO_2_, CO) have their “fingerprints”.

Thus, all final configurations presented in this section use a SiO_2_ layer as dielectric material, deposited on the Pt heater, applying the methodology presented in Section 2.

**Figure 8:**
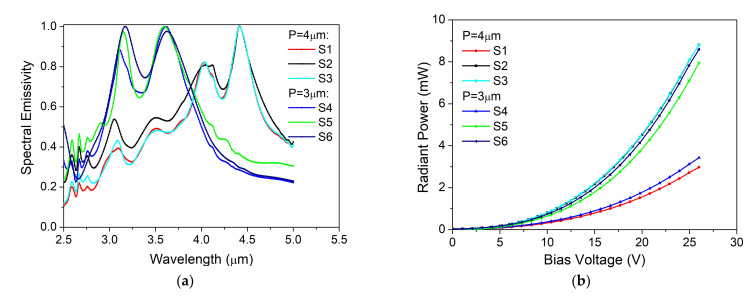
Examples of optical and electrical capabilities for six different IR emitter configurations (S1–S6): (**a**) spectral emissivity for a 20 V bias voltage; (**b**) the radiant power as a function of applied bias voltage.

Figure 8a shows the normalized spectral emissivity for six different combinations of MPA and heater configurations with different gold resonator periods and diameters and different numbers of meanders and types of pads (Table 2). It can be observed that the change of gold disk period is responsible for the tunability of the IR emitter. Thus, the system with a period of 4 μm shows two peaks, at 4.41 and 4.04 μm, in the spectral range where carbon dioxide absorbs light, while the system with a period of 3 μm has also two main peaks in the spectral range but where the methane absorbs the electromagnetic radiation (around 3.6 and 3.15 μm). The background emissivity was between 0.2 and 0.4 for all samples presented here. As can be seen in Table 2, a good reproducibility of the sample configurations was obtained. The S1, S2, and S3 samples with the same MPA configuration have approximately the same emission peaks. The small shifts in emission spectra are the results of the type of heater configuration employed in each system. The measured radiant power values for all studied IR systems varied from sample to sample, depending on the structure of the heater and the applied bias voltage.

**Figure 9:**
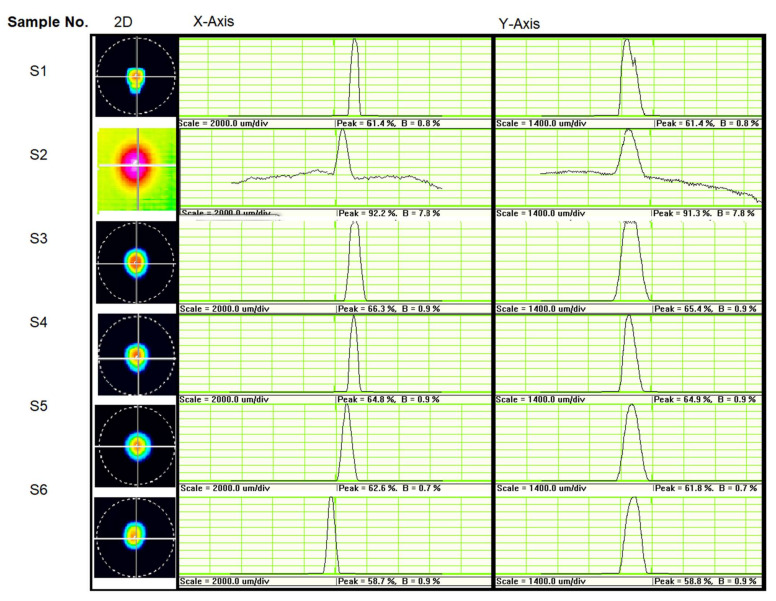
The 2D beam profile with a profile on the X- and Y-axis corresponding to a bias voltage of 14 V applied on selective emitter pads.

The radiant power trend is similar to the one of heaters as one can see in Figure 6b and Figure 8b, with the highest values obtained for the two-meander configurations. The maximum radiant power value obtained for the selective IR emitter was 7.3 mW in comparison with 12.5 mW obtained for the heater without the MPA attached, when a bias voltage of 24 V was applied. The current voltage curve of the IR system showed a behavior similar to that of the microheaters. The beam profile quality was also measured using an IR camera, and the results are presented in Figure 9 The beam profiles were measured at a distance of 96 cm between the IR emitter and the sensitive area of the camera when a bias voltage of 14 V was applied. All six configurations tested and presented here showed narrow and Gaussian beam profiles on both axes, X and Y. From the 2D beam profile can be seen the emitter uniform emissivity, concentrated in the center of the samples.

**Figure 10:**
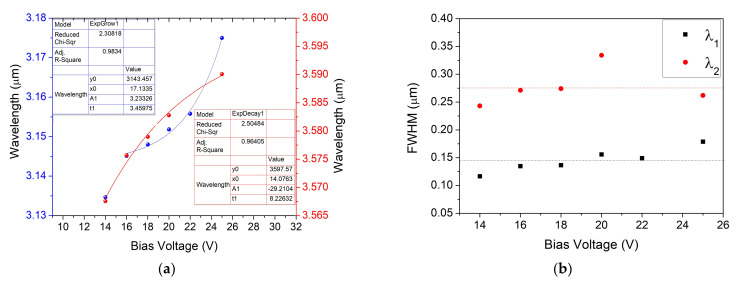
Sample S6 (**a**) wavelength spectral shift and (**b**) FWHM, variation for the main peaks depending on the bias voltage applied on the IR emitter pads. Peak characteristics: λ_1_ = 3.152 nm; λ_2_ = 3.579 nm, with corresponding FWHM_1avg_ = 0.145 nm ± 0.021 mm and FWHM_2avg_ = 0.277 mm ± 0.034 mm.

In order to determine the damage threshold of the systems, the radiant power change was measured for voltages between 0 and 32 V, and in the case of the S6 system, a spectral shift to larger wavelengths for voltages between 14 and 32 V was noticed (Figure 10). This shift of up to 50 nm, obtained by changing the voltage values, gives the user the possibility to tune the system around the absorption central peak of targeted gas molecules. The mean FWHM (full width half maximum) values obtained for the system with two meanders, large pads, a diameter of 1.6 μm, and a period of 3 μm were around 200 nm for the highest emitted peak and around 275 nm for the second one, showing the feasibility of this system to be used for gas detection applications.

### 3.3. Sensor Functionality Test

**Figure 11:**
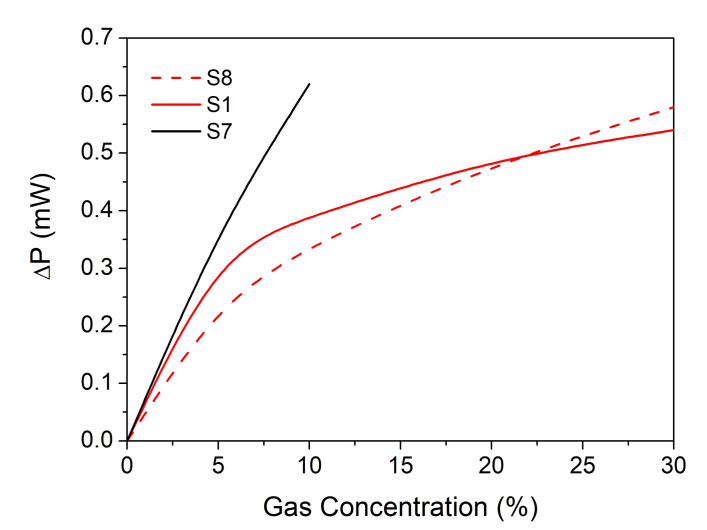
Changes in radiant power when different concentrations of CO_2_ gas are introduced in the sampling chamber.

To prove the proposed sensor functionality, a gas chamber was developed (Figure 2, Section 4). The changes in optical power emitted by the narrow-band IR source that reaches the detector was measured when a toxic gas stream was introduced into the measurement chamber. By changing the ratios of toxic gas (test gas: CO_2_, CH_4_, CO) in the inert gas (nitrogen, N_2_ 5.0, purity 99.999%), keeping the flow rate constant at 1000 L/min, it was possible to modify the concentrations of the introduced toxic gas. For example, to detect CO_2_ gas molecules, based on our findings from Section 3.2, we tested various IR emitters, which proved good emissivity properties (Figure 8a) in the spectral range where CO_2_ molecules absorb IR radiation. Figure 11 shows the variation of the optical power measured by the detector function on the CO_2_ concentration for three samples. The sensor that proved to be most sensitive to CO_2_ was S7. To demonstrate the proof of concept, preliminary results were obtained for CO sensing, using an S9 structure, and for CH_4_, using an S6 structure.

## 4. Conclusions

Different IR selective sources, emitting in the spectral range of 3 ÷ 5 μm, were tested, and their feasibility for toxic gas sensing was proved in this paper. Four different configurations for the microheater and several configurations for the metamaterial absorber were tested to demonstrate the high selectivity of the emitter. From the experimental data, narrow emission peaks (FWHM up to 0.150 μm), corresponding to the CO_2_, CH_4_, and CO absorption bands, with a radiant power of a few mW were obtained. From our knowledge, this is one of the few selective IR emission sources based on the integration of a plasmonic metasurface structure specifically configured to enhance the absorption of radiation over a narrow wavelength range in the infrared spectral range with a broadband thermal source via an amorphous silicon layer in order to obtain a narrow-spectrum IR source. The narrow emission band with FWHM lower than that recently reported in [22] can ensure a high selectivity of the proposed gas detection system.

The results show that the best emissivity is obtained if the sensor has the following configuration: (i) substrate: 500 µm thick fused silica wafer; (ii) heater that generates a uniform broadband thermal radiation and acts as a backplane for the MDM metasurface: 80 nm of platinum deposited by electron beam evaporation method and patterned with two meanders and large contact pads to obtain a resistive heater; (iii) dielectric: 100 nm of SiO_2_ deposited at 700 °C (which ensures that the layer will not be damaged by high temperatures released by the heater); (iv) metasurface composed of Au nanodisks, with diameters between 1.2 and 1.6 μm and a disk periodicity of 3 or 4 μm, depending on the targeted gas; and (v) a thin layer of amorphous silicon that improves the spectral characteristics of the IR emitter. It was demonstrated that, applying a bias voltage of 24 V to the structure with this configuration, two intense (3.15 and 3.6 µm) and narrow (FWHM equal to 150 nm and respective 250 nm) emission spectra, with a corresponding radiant power of 9 mW, are obtained. This emission matches the absorption features of methane molecules. Additionally, for a similar heater configuration, but a period of 4 µm and a disk diameter of 2.2 µm, two emission peaks at 4.04 and 4.41 µm, having an FWHM of around 275 nm, covering the absorption pattern of carbon monoxide and dioxide gas molecules, are obtained. Therefore, one conclusion that can be drawn from this study is that just by changing the period of MPA gold disks, the emission peak can be easily changed, and by changing the heater meanders and contact pad configuration, the intensity of the emission peaks can be improved.

Moreover, for the MPA structure with a disk diameter of 1.6 µm, by changing the bias voltage, a shift of a few tens of nm is obtained around the central emission wavelength, allowing fine optimization for gas sensing applications.

We demonstrated that the presented structures can be employed in the development of gas sensing systems. To this end, a specifically designed gas chamber was built, and the changes in optical power that reaches the photodetector were analyzed to determine the sensitivity of the structures in the presence of specific gasses. All measurements were performed by mixing the target gas with an inert one in different concentrations. By gradually increasing the concentration of the target gas, we observed a decrease in the optical power that reaches the detector. The best result sensitivity was obtained for the detection of small concentrations of CO_2_ with a structure composed of resistive IR source in two-meander configuration integrated with a metasurface having elements of 1.2 µm diameter and 3 µm period. The best sensitivity for CO detection was obtained for a structure with two-meander configuration integrated with a metasurface having nanodisks with a 1.3 µm diameter and a 4 µm period. The optimal results for methane detection were obtained for a structure with two-meander configuration integrated with a metasurface having nanodisks with a 1.6 µm diameter and a 3 µm period.

Preliminary results were obtained for the detection of CO and CH_4_.

The proposed sensor system can be used in gas sensing application for security and safety, and also for remote sensing, in the detection and monitoring of pollutants or greenhouse gases.

## Figures and Tables

**Table 1:** Geometrical parameters for the different microresonators’ arrays, their absorption maxima, and the corresponding gases having the fingerprints at the metamaterial absorption peak.

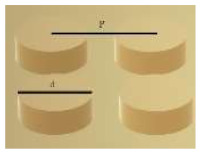	**No.**	**Disk Diameterd (µm)**	**Disk Period** **P (µm)**	**Absorption Peak** **A (µm)**	**Type of Gas**
	1	1.6	3	3.6	CH_4_/formaldehyde
	2	2	3	3.4/5.1	CH_4_/NO
	3	2.1	3	6.3/3.3	NO_2_/CH_4_
	4	1.5	4.5	5.11	NO
	5	1.3	4	4.46/4.95	CO
	6	1.4	4	5	NO/CO
	7	1.7	4	4.15/5.6	CO_2_/NO
	8	2.2	4	4.15	CO_2_

**Table 2:** IR emitter spectral performances with the corresponding gas molecules having absorption peaks in the emitter emission range.

Sample	Meanders	Pads	d (μm)	P (μm)	Spectral Range (μm)	Main Peak (μm)	Second Peak (μm)	Third Peak (μm)	Corresponding to Gas Molecule Abs. Peak
S1	3	Narrow	2.2	4	3.9...4.16 4.25...4.55	4.41	4.03	3.09	NO_2_, CO_2_
S2	2	Narrow	2.2	4	3.9...4.2 4.37...4.43	4.41	4.04	3.05	NO_2,_ CO_2_
S3	2	Large	2.2	4	3.91...4.17 4.25...4.5	4.41	4.04	3.09	NO_2_, CO_2_
S4	3	Large	2	3	3.1...3.3	3.17	3.62	-	CH_4_
S5	2	Large	2.1	3	3.08...3.23	3.61	3.11	-	CH_4_
S6	2	Large	1.6	3	3.05...3.2	3.6	3.15	-	CH_4_
S7	2	Narrow	1.2	3	3.1...3.2 3.9...4.35	3.15	4.05	4.3	CH_4_, CO_2_
S8	3	Narrow	1.4	4	4.2…4.6	4.4	6	-	CO_2_
S9	2	Large	1.3	4	4.3…4.8	4.5	6.1	-	CO

## Data Availability

The data presented in this study are available on request from the corresponding author.
